# Clinical practice guidelines for patients with gastrointestinal stromal tumor in Taiwan

**DOI:** 10.1186/1477-7819-10-246

**Published:** 2012-11-15

**Authors:** Chun-Nan Yeh, Tsann-Long Hwang, Ching-Shui Huang, Po-Huang Lee, Chew-Wun Wu, Ker Chen-Guo, Yi-Yin Jan, Miin-Fu Chen

**Affiliations:** 1 Department of General Surgery, Chang Gung Memorial Hospital, Chang Gung University, #5, Fu-Hsing Street Kwei-Shan, Taoyuan, Taiwan; 2 Department of Surgery, National Taiwan University Hospital, Taipei, 100, Taiwan; 3 Department of Surgery, Taipei Veteran General Hospital, Taipei, 100, Taiwan; 4 Department of Surgery, Yuan’s General Hospital, Kaohsiung City, Taiwan

**Keywords:** Guidelines, Gastrointestinal stromal tumors, Imatinib, Targeted, Treatment

## Abstract

For many years, the understanding of gastrointestinal stromal tumors (GISTs), which are the most common mesenchymal tumors of the gastrointestinal tract, has been very limited. However, it is now possible to provide a more precise definition through the use of pathology classification and molecular techniques. Coupled with the advancement of clinical practice, especially the development of targeted therapy, there is now a much better insight into its treatment. At present, organizations such as the National Comprehensive Cancer Network in the USA and the European Society for Medical Oncology in Europe have established a consensus and drawn up guidelines for the diagnosis, treatment, and follow-up of GISTs.

With experts coming from various districts in Taiwan and combining the most recent clinical data and experiences, the Taiwan Surgical Society of Gastroenterology drafted the first national GIST treatment guidelines after a consensus meeting in 2007. Following subsequent advances in GIST diagnosis and treatment, further revisions and modifications have been made to the original guidelines. We present here the updated consensus and recommendations of the Taiwan Surgical Society of Gastroenterology for the diagnosis and treatment of GIST. We hope these guidelines can help enhance the quality of diagnosis, treatment, and care of patients with GIST in Taiwan.

## Review

Gastrointestinal stromal tumors (GISTs) are the most common mesenchymal tumor of the gastrointestinal tract, and account for 5% of all sarcomas [1]. Although GISTs are relatively rare tumors, the reported incidence has increased since the early 1990s, owing to increased awareness and appropriate diagnosis of this tumor type. In Taiwan, the annual incidence of GIST is 13.74 per million populaton [2], consistent with studies from other countries ,which show annual incidences of 11 to 19.6 per million population [3-7]. In general, only complete resection can lead to cure, although recurrence is common after surgery. Before the advent of targeted therapies, the prognosis for advanced GISTs was poor, owing to their inherent resistance to conventional chemotherapy and radiotherapy [8]. The identification of thje signal-transduction pathway associated with the development of GISTs and the use of molecular targeted therapies, such as imatinib mesylate (Gleevec/Glivec; Novartis Pharmaceuticals, Basel, Switzerland), have dramatically improved the survival and quality of life of patients with GISTs over recent years.

In western countries, several organizations including the National Comprehensive Cancer Network (NCCN) and the European Society of Medical Oncology (ESMO) have published updated guidelines for the diagnosis and management of GIST [9-11]. In Taiwan, the Taiwan Surgical Society of Gastroenterology (TSSG) drafted the first national GIST treatment guidelines after a consensus meeting involving experts from across the country in 2007 (unpublished data). Following subsequent advances and developments, the group of experts conducted a series of meetings to review more recent evidence and made modifications to the original guidelines. This review presents the updated consensus and recommendations of the TSSG as a basis for guidelines for the diagnosis and treatment of patients with GIST in Taiwan. Table 1 shows the levels of evidence [I to V] and grades of recommendation [A to D], as used by the American Society of Clinical Oncology [12].

**Table 1:** Levels of evidence and grades of recommendation

**Level**	**Source of evidence**	**Grade**	**Grade of recommendation**
I	Meta-analysis of multiple well-designed, controlled studies; randomized trials with low rates of false-positive and low false-negative errors (high power)	A	Evidence rated as level I or consistent findings from multiple studies at levels II, III, or IV
II	At least one well-designed experimental study; randomized trials with high rates of false-positive and high false-negative errors (low power)	B	Evidence at levels II, III or IV, and generally consistent findings
III	Well-designed, quasi-experimental studies such as non-randomized, controlled, single-group, preoperative and correlation descriptive studies, and case studies	C	Evidence at levels II, III or IV, but inconsistent findings
IV	Well-designed, non-experimental studies such as comparative and correlation descriptive studies, and case studies	D	Little or no systematic empirical evidence
V	Case reports and clinical examples		

### Disease background

As first reported in 1998, 95% of GISTs are immunohistochemically positive for the receptor tyrosine kinase KIT (also known as CD117) [13]. In addition, Hirota and colleagues found that in most GISTs, the KIT protein has been mutated, leading to constitutive activation of the kinase [13,14]. It is now known that 70 to 80% of GISTs harbor a KIT mutation. Most KIT mutations occur in the juxtamembrane domain encoded by KIT exon 11, and some have been detected in the extracellular domain encoded by exon 9. KIT mutations have also been identified in the tyrosine kinase domain (exons 13 and 17), although these are rare [15,16]. A subset of GISTs that lack KIT gene mutations harbor an activating mutation in the gene encoding platelet-derived growth factor receptor alpha (PDGFRA) [3]. KIT and PDGFRA mutations are mutually exclusive, and are associated with distinct clinicopathologic features. For example, GISTs with KIT exon 9 mutations are often located in the small bowels, whereas PDGFRA-mutated GISTs are commonly found in the stomach [17,18]. The PDGFRA mutation is present in about 5% of GISTs in western patients, but the mutation rate is much lower in Taiwanese patients (about 1%) [3,19]. About 10 to 15% of GISTs do not have a detectable mutation in either KIT or PDGFRA, and are often referred to as ‘wild-type’ GISTs. KIT remains a key diagnostic marker for this tumor type, and mutant KIT and PDGFRA proteins have become crucial therapeutic targets in GISTs.

GISTs are predominantly found in middle-aged to older adults, and are extremely rare in patients younger than 30 years [20]. The median age at diagnosis has been reported to be in the range of 63 to 69 years [6,7,21]. The most common primary sites for GISTs are the stomach (60%) and small intestine (30%), with the duodenum (5%), colorectum (< 5%), and esophagus and appendix (<1%) being less common sites [22,23]. Recurrence after resection is predominantly intra-abdominal, and the liver is the most common site of recurrence in both patients with a primary tumor and those with metastatic disease at presentation [1].

In general, patients with suspected GIST may present with various symptoms, including, but not limited to, early satiety, fatigue secondary to anemia, intraperitoneal hemorrhage, intra-luminal gastrointestinal bleeding, or abdominal discomfort from pain or swelling. Some patients may present with an acute abdomen as result of tumor rupture, gastrointestinal obstruction, or appendicitis-like pain, which requires immediate medical attention.

### Diagnosis

#### Clinical diagnosis

We recommend that potentially resectable GISTs of any size, other than tumors found in the stomach, should be referred to a general surgeon for resection. Suspected gastric nodules 20 mm or larger in size should be surgically resected, because, if diagnosed as GIST, will imply a higher risk [9-11]. Nodules smaller than 20 mm, if diagnosed as GIST, may be low-risk, and their clinical significance remains questionable. However, we recommend that patients with suspected gastric GIST smaller than 20 mm should be referred for resection if any of the following is present: 1) nodule with irregular margin, signs of ulceration or bleeding, or an increase in size during follow-up; 2) presence of cystic change, necrosis, heterogeneous echogenecity, or lobulation, or if there is poor patient compliance with follow-up; or 3) diagnostic confirmation of GIST through fine-needle aspiration biopsy (FNAB) or if it is a KIT-positive tumor. When there is a strong suspicion of gastric GIST based on endoscopic ultrasonography without histological confirmation, surgical resection or close follow-up may be considered [10]. Percutaneous biopsy is not encouraged, because it is associated with a risk of hemorrhage and intra-abdominal tumor dissemination [10].

#### Molecular pathologic diagnosis

Pathologically, the diagnosis of GIST can be confirmed by morphology and immunohistochemistry. GISTs have a characteristic immunohistochemical profile useful for diagnosis [24]. Approximately 95% of GISTs are positive for KIT, which makes KIT positivity a key defining feature of GIST, but alone it may not be sufficient to allow diagnosis. Other commonly expressed markers include CD34 antigen (70%), smooth muscle actin (SMA; 30 to 40%), desmin (<5%), and S100 protein (~5%) [24]. A recently described antibody against Discovered on GIST-1 (DOG1) has been reported to be as sensitive as KIT in diagnosing GIST, but DOG1 is expressed only in about 30% of KIT-negative GISTs, limiting its use in this setting [25].

**Figure 1:**
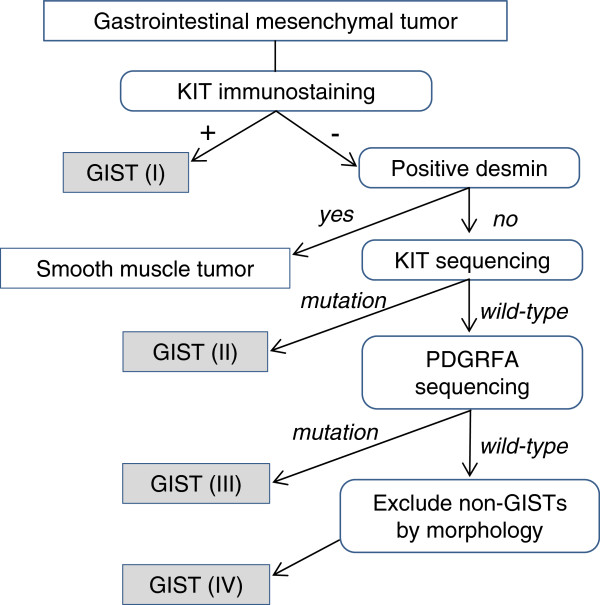
**Recommended algorithm for the molecular diagnosis of gastrointestinal stromal tumor by immunohistochemistry and mutation analysis.** Adapted from Miettinen *et al*. [25]. GIST, gastrointestinal stromal tumor; PDGFRA, platelet-derived growth factor receptor-α.

In the small proportion of GISTs (about 5%) that are KIT-negative, or in patients with an unclear diagnosis or atypical morphology or clinical features, mutational analysis for known mutations involving the *KIT* and *PDGFRA* genes should be performed to confirm a diagnosis of GIST [26]. Figure 1 shows an algorithm for the diagnosis of GIST based on immunochemistry and mutational analysis.

#### Imaging diagnosis and follow-up

Imaging is a useful diagnostic for confirming and staging GISTs and follow-up. Currently, all patients with suspected GIST should undergo abdominal/pelvic computed tomography (CT) scanning with contrast and/or magnetic resonance imaging (MRI). CT scanning is preferred over MRI if only one imaging procedure can be performed. CT is also a sensitive and specific method to assess the response of GISTs to imatinib treatment [27]. When used for response evaluation, CT scan should be based on a tailored standardized protocol, and the assessment of therapeutic effect should include changes in tumor size and density. ^18^F-fluorodeoxyglucose (FDG)-positron emission tomography (PET) has also been shown to be sensitive in detecting early response and to be useful in assessing tumor response [9,28]. When CT scans cannot be accurately evaluated, findings from FDG-PET can be used to support the evaluation of the CT scan reading. FDG-PET evaluation for treatment response should be based on the uptake intensity of ^18^-FDG.

#### Risk stratification of primary GIST

Accurate risk classification of GISTs has become increasingly important, owing to emerging adjuvant systemic treatment. All GISTs are considered to have some malignant potential, and there are several systems such as the National Institute of Health (NIH) criteria, the Huang modified NIH criteria, and the Armed Forces Institute of Pathology (AFIP) criteria commonly used to determine the risk of recurrence. Joensuu *et al*. found in an analysis of pooled population-based cohorts that all three risk-stratification schemes were reasonably accurate at predicting outcome. Those authors developed new prognostic contour mapsbased on non-linear modeling of tumor size and mitotic count, which might be useful for estimation of individualized outcomes [29]. Of the three risk-stratification systems, the AFIP criteria are considered the most informative for predicting the survival of localized primary GISTs [22]. Thus the tumor size, mitotic count per 50 high-power fields (HPFs) and tumor location are considered the three most important prognostic factors for prediction of GIST recurrence.

#### Recommendations for diagnosing GIST

Below are the general recommendations of the TSSG for the diagnosis of GIST.

In general, patients should be managed by a multidisciplinary team (MDT) with expertise in sarcoma. Shared decision-making between the MDT and the patient is recommended.

Initial investigations in patients with suspected GIST should include history- taking and physical examination, appropriate imaging of the abdomen and pelvis using CT scan with contrast and/or MRI, chest imaging, endoscopic ultrasonography, and endoscopy, if not previously performed.

All patients with potentially resectable GISTs, except those with tumors in the stomach, should be referred for surgical resection.

Patients with suspected gastric GIST 20 mm or larger should receive surgical resection. It is strongly suggested that those with suspected gastric nodules of less than 20 mm in size are also referred tfor resection if any of the following is present: 1) nodule with signs of irregular margin, ulceration, bleeding or increase in size during follow-up; 2) presence of cystic change, necrosis, heterogeneous echogenecity, lobulation, poor patient compliance with follow-up; or 3) diagnostic confirmation of GIST by FNAB or presence of KIT-positive tumor.

When the diagnosis of gastric GIST is strongly suspected based on endoscopic ultrasonography but without histological confirmation, surgical resection or close follow-up may be considered. Percutaneous biopsy is not encouraged.

Mutation analysis should be performed in KIT-negative patients and in patients with an unclear diagnosis or atypical clinical features.

For imaging diagnosis and follow-up, CT scan is preferred over MRI if only one imaging procedure can be performed.

When used for response evaluation, CT scan should be based on a tailored standardized protocol, and the assessment of therapeutic effect should include changes in tumor size and density.

FDG-PET can be used to support the CT scan reading when the CT scan cannot be accurately evaluated. FDG-PET evaluation for treatment response should be based on the uptake intensity of ^18^FDG.

#### Surgical treatment

Surgery remains the mainstay of therapy for patients with primary GIST and no evidence of metastasis [9-11]. The goals of surgery include complete resection, avoidance of tumor rupture, and intra-operative staging to exclude metastatic disease.

The preferred resection margin is 10 mm grossly. Lymph-node dissection is usually unnecessary because lymph-node metastases are rare with GIST and indeed, with sarcomas in general [30]. Preoperative biopsy is not recommended for potentially resectable GIST, and is associated with slight risks [9]. GISTs may be soft and fragile, and biopsy may cause hemorrhage and increase the risk of the tumor seeding. It is often difficult to make a definitive diagnosis with FNAB, and a core needle biopsy may be inconclusive if a necrotic or hemorrhagic portion of the tumor is sampled. Therefore, postoperative pathology assessment is crucial to confirm the diagnosis after removal of any suspected GIST.

Even after complete resection of primary localized GIST, some patients are at high risk of tumor recurrence [1,8]. In a Taiwanese study of 85 patients with GIST who had undergone complete resection, the 5-year disease-free survival (DFS) and overall survival (OS) rates were 43.7% and 50.5%, respectively [31]. Similar survival rates in the range of 40 to 65% have been reported in other studies [1,32-36].

The role of surgery in patients with metastatic GIST after treatment with imatinib has been evaluated in several studies. Medical treatment of metastatic GIST with imatinib alone usually does not result in complete response. Furthermore, responses are not maintained indefinitely, and resistance usually develops. Surgery after imatinib treatment has been shown to prolong progression-free survival (PFS) and OS in Taiwanese patients with responsive tumors or local progression [37]. Furthermore, surgery for selected responsive lesions may play a role in preventing potential development of secondary mutations, which is the main reason for resistance and eventually progression [37]. Similarly, Raut *et al*. found that surgery prolonged OS in patients with advanced GIST exhibiting stable disease or limited progression on imatinib therapy [38]. Surgery did not result in any survival benefit in patients with generalized disease progression [38].

Therefore, the combined use of surgery and imatinib treatment may be beneficial for selected patients with metastatic GIST if the disease is responsive to imatinib, or if progression is localized. Surgery is not indicated in systemic progressive disease, unless for complications such as obstruction, bleeding, or perforation [9].

#### Recommendations for surgical treatment

Hence, the recommendations for surgical treatment are as follows.

The surgical goals for resectable GIST include complete resection, avoidance of tumor rupture, and intra-operative staging to exclude metastatic disease**.** The preferred resection margin is 10 mm grossly. Lymph-node dissection is unnecessary.

Combined use of surgery with imatinib treatment may benefit selected patients with metastatic GIST that is responsive to imatinib and exhibits only localized progression [level of evidence IIIB].

Surgery with imatinib treatment is not indicated for systemic progressive disease unless for complications such as obstruction, bleeding, or perforation.

Biopsy is not recommended for potentially resectable GIST.

#### Medical treatment

##### Adjuvant treatment

Postoperative adjuvant chemotherapy with conventional cytotoxic agents has not generally been recommended for GIST because these agents are ineffective against the cancer [8]. In view of the likelihood of tumor recurrence after surgical resection, several studies have investigated the role of adjuvant imatinib treatment in GIST, and suggested that it is useful in patients at significant risk of recurrence after tumor resection [39-41]. Imatinib is an oral agent that is a selective molecular inhibitor of the KIT, PDGFRA, ABL, and BCR-ABL tyrosine kinases. Imatinib was first used for chronic myelogenous leukemia, for which it proved to be safe and to be capable of achieving complete hematological response in nearly all patients through inhibition of the BCR-ABL oncoprotein [42]. The efficacy of imatinib against metastatic GIST was first shown in 2000 [32], and subsequently confirmed in phase II and phase III trials in metastatic disease [43-46].

The American College of Surgeons Oncology Group (ACOSOG) first conducted an open-label, multicenter, phase II trial (Z9000) to evaluate the efficacy of postoperative imatinib in 106 evaluable patients with primary GIST who were at high risk for recurrence (tumor size ≥100 mm, tumor rupture, or <5 peritoneal metastases) [39]. The results showed that postoperative imatinib 400 mg daily for 1 year prolonged recurrence-free survival (RFS) after complete resection, and was also associated with improved OS compared with historical controls. A subsequent ACOSOG phase III, double-blind, randomized trial (Z9001) in patients with KIT-expressing GIST of at least 30 mm in size confirmed that 1 year of adjuvant imatinib (400 mg/day) significantly improved 1-year RFS rates after complete resection compared with placebo (98% versus 83%, *P*<0.0001) [40]. Based on the Z9001 phase III data, imatinib (400 mg/day) has been approved by the US Food and Drugs Administration (FDA) for the adjuvant treatment of adult patients after complete surgical removal of KIT-positive GISTs [47]. A recent randomized, open-label, phase III study (SSGXVIII/AIO) evaluated adjuvant imatinib therapy for 3 years compared with 1 year in patients with KIT-positive GIST removed by surgery who were at high risk of recurrence (tumor size >100 mm or tumor with a mitotic rate of >10 mitoses/50 HPFs or tumor size >50 mm and a mitotic rate of >5 mitoses/50 HPFs or tumor rupture) [41]. The results showed that 3 years of adjuvant imatinib significantly improved the 5-year RFS (65.6% vs 27.9%, p<0.001) and OS (92.0% versus 81.7%, *P*<0.02) compared with 1-year imatinib therapy. The role of longer-term treatment and the optimal duration of adjuvant imatinib remain to be determined by further studies. Based on current clinical evidence, adjuvant imatinib is recommended for intermediate (≥60 ,m and <100 mm) to high-risk primary GISTs (mitotic count >5 mitoses/50 HPFs; size >50 mm; non-gastric location; and tumor rupture), and treatment duration and criteria usually follow the guidelines for national health insurance reimbursement in Taiwan.

##### Recurrent or metastatic disease

In agreement with the NCCN and ESMO guidelines, we recommend that imatinib should be used as first-line therapy for unresectable, recurrent, or metastatic GIST [9-11]. Recurrence is common after surgical resection of primary GIST, and the site of first recurrence is typically within the abdomen and involves the peritoneum, liver or both. Before the era of imatinib, treatment options for patients with recurrent or metastatic disease were limited because of the poor response of GISTs to conventional chemotherapy and radiotherapy, making their outlook very poor. The median time to recurrence after resection was approximately 2 years [1,36].

The clinical benefit of imatinib has been shown in western and East Asian patients with advanced unresectable or metastatic GIST. The phase II randomized trial (B2222) and subsequent long-term follow-up analysis showed that imatinib 400 mg or 600 mg daily induced a sustained objective response in more than half of patients with advanced unresectable or metastatic GIST, extending the median survival to 57 months [43,44]. Similar benefits were achieved in patients who had objective responses and patients who had stable disease [44]. The optimal dose of imatinib was further investigated in two multicenter, randomized phase III trials, (S0033 and the European Organisation for Research and Treatment of Cancer (EORTC) study 62005), which compared standard-dose (400 mg daily) with high-dose (800 mg daily) imatinib, with the option for patients whose disease progressed on the standard dose to cross over to the high dose [44,45]. Results from the trials confirmed the effectiveness of imatinib as primary therapy for advanced or metastatic GIST, but both the S0033 trial and the longer-term follow-up analysis of the EORTC 62005 did not show a significant advantage for the high-dose treatment [42,46]. A subsequent meta-analysis of 1,640 patients with advanced GIST from the S0033 and EORTC 62005 trials (MetaGIST) showed a small PFS advantage of high-dose imatinib, essentially for patients with KIT exon 9 mutations, but no OS advantage [47].

Studies in Taiwanese patients confirmed the survival benefits of imatinib in patients with advanced GIST. An early study in 22 patients with advanced GIST showed that patients treated with imatinib 400 mg daily had a significantly longer post-recurrence survival and OS compared with those not treated with imatinib [48]. The partial response rate was 68.2%, and this was similar for patients with KIT exon 9 and those with KIT exon 11 mutations. A longer-term study of 171 Taiwanese patients with advanced or metastatic GIST treated and followed up within a 10-year period (median follow-up 33.6 months) confirmed that imatinib induced a sustained objective response in more than half of the patients (57.4%) [49,50]. The median PFS and OS rates were 37.6 and 71.0 months, respectively. Similar to the MetaGIST study, the clinical benefit of imatinib was significantly higher in patients harboring KIT exon 11 mutations than in those harboring KIT exon 9 mutations (93.3% versus 61.9%, *P* = 0.0005), suggesting that patients with KIT exon 9 mutations may benefit from escalating the imatinib dose to 800 mg daily [47].

##### Neoadjuvant treatment

Neoadjuvant imatinib treatment may reduce tumor size or spread, and enable patients with previously unresectable GISTs to undergo surgical resection. Two phase II trials have evaluated the efficacy and safety of preoperative imatinib for GIST. The Radiation Therapy Oncology Group (RTOG) prospective phase II study (RTOG 0132) was the first to evaluate the neoadjuvant use of imatinib 600 mg/day for patients with advanced primary GIST (n = 30) and the preoperative use of imatinib in patients with potentially operable metastatic/recurrent disease (n = 22) [51]. Response rates after 8 weeks of preoperative imatinib in accordance with the Response Evaluation Criteria in Solid Tumors (RECIST) system were 7% partial and 83% stable disease., while the corresponding response rates in patients with recurrent or metastatic disease were 4.5% and 91%, respectively. The 2-year PFS rates were 83% for patients with primary GIST and 77% for those with recurrent or metastatic GIST, and the estimated OS rates were 93% and 91%, respectively. Complications of surgery and imatinib toxicity were reported to be minimal.

Another phase II trial randomized 19 patients with GIST undergoing surgical resection to receive 3, 5, or 7 days of preoperative imatinib 600 mg daily. All patients received postoperative imatinib for 2 years [52]. The response rates as assessed by ^18^FPG-PET and dynamic CT were 69% and 71%, respectively. Median DFS of patients treated with surgery and imatinib was 46 months, and imatinib did not affect surgical morbidity compared with an imatinib-naive cohort. The true survival benefit of preoperative imatinib could not be determined because all patients received postoperative imatinib for 2 years in both trials. The optimal duration of preoperative imatinib also needs to be defined.

Neoadjuvant imatinib should be considered for patients with marginally resectable tumors or resectable GISTs who have a risk of significant morbidity [9]. Neoadjuvant imatinib can also be considered for patient with primary localized GIST whose tumors are deemed unresectable. The decision to use preoperative therapy for patients with resectable primary or locally advanced GIST should be made based on clinical judgment and on an individual basis. When neoadjuvant treatment is considered, progression and response of tumors before and during the treatment should be assessed carefully by an MDT, based on CT (with optional MRI) scan and/or PET scan results. Early assessment of tumor response is recommended, so that surgery is not delayed in the case of non-responding tumors. Continuous imatinib treatment should be considered for patients with GISTif administered before resection and if an objective response is obtained.

##### Second-line treatment of advanced GISTs

Regarding disease progression during imatinib therapy, resection of the progressing lesion should be considered if it is feasible and progression is limited [38]. For patients with limited progressive disease, or those with generalized progressive disease and good performance status (0 to 2), options include continuation of imatinib at the same dose, dose escalation as tolerated (600 to 800 mg/day), or switching to sunitinib after failure on imatinib [9-11]. Radiofrequency ablation and chemoembolization can also be considered for patients with limited progressive disease [9,10]. Treatment response should be reassessed carefully by CT or PET scan.

Sunitinib is an oral multi-targeted tyrosine kinase inhibitor with activity against KIT and PDGFRA as well as other pathways that may be relevant in GIST, such as vascular endothelial growth factor receptor [53,54]. Sunitinib has received multinational approval for the treatment of GIST after failure of imatinib due to resistance or intolerance, based on the results of an international, randomized, double-blind, placebo-controlled phase III trial [55]. The trial results showed that sunitinib 50 mg daily in a 4/2 schedule (4 weeks on and 2 weeks off treatment) significantly prolonged the time to progression compared with placebo in patients with advanced GIST who were resistant or intolerant to imatinib (27.3 weeks versus 6.4 weeks, *P*<0.0001). Continuous daily dosing with sunitinib 37.5 mg daily has also been reported to be active, and compares favorably with the 4/2 schedule [56]. A retrospective study in Taiwanese patients with imatinib-resistant or imatinib-intolerant GIST showed that sunitinib induced a sustained clinical benefit in 65.2% of the patients, and the median PFS and OS were 8.4 and 14.1 months, respectively [57].

There are also some newer agents that are under investigation for the treatment of GISTs. These include the second-generation tyrosine kinase inhibitors nilotinib, dasatinib, and sorafenib [9,10].

##### Recommendations for medical treatment

Adjuvant imatinib treatment should be considered in high-risk patients after complete or incomplete resection of primary tumor [level of evidence IIB].

Neoadjuvant imatinib should be considered for patients with: 1) marginally resectable tumors or resectable GISTs, who have a risk of significant morbidity; or 2) primary localized GIST, whose tumors are deemed unresectable.

When neoadjuvant treatment is considered, progression and response of tumors before and during the treatment should be assessed by the MDT, using CT (with optional MRI) and/or PET scans.

Imatinib 400 mg daily should be initiated as first-line therapy for recurrent or metastatic GIST [level of evidence IA]. Higher doses, up to 800 mg daily, should be considered for patients with exon 9 KIT mutation [IIIA].

In case of limited disease progression during imatinib therapy, resection of progressing lesion should be considered if feasible. Radiofrequency ablation and chemoembolization can be considered to control limited progression. Imatinib should be continued at the same dose or at an increased dose (600 to 800 mg/day) [level of evidence IIIB], as tolerated. After failure on imatinib, sunitinib can be considered as second-line therapy [IIB].

In patients with generalized progressive disease and performance status 0 to 2, imatinib should be administered at a higher dose (600 to 800 mg/day), as tolerated [level of evidence IIIB], and sunitinib should be considered after failure of imatinib [IIB]. Treatment response after progression should be reassessed carefully by CT or PET scan.

## Conclusion

**Figure 2:**
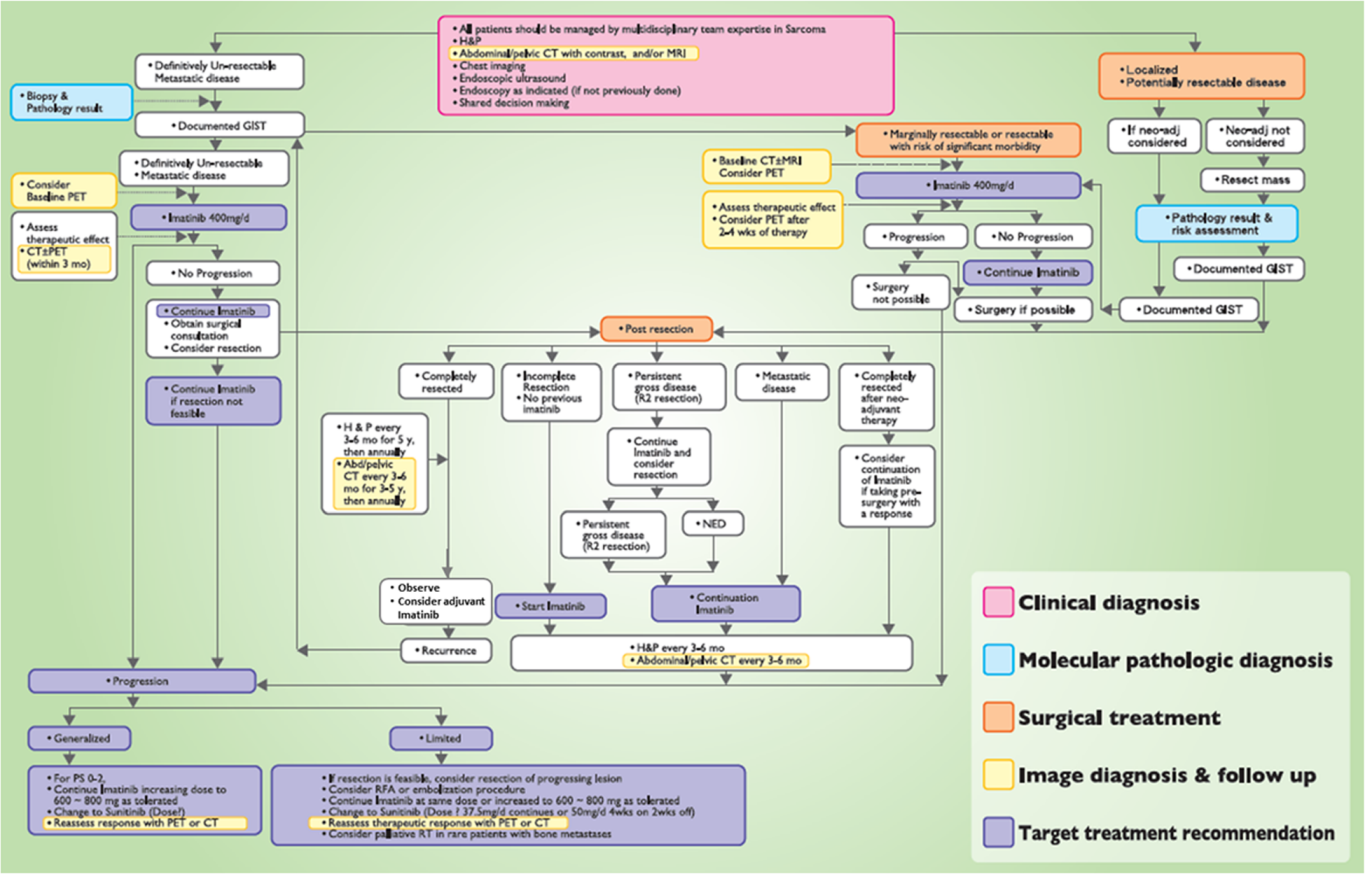
The treatment procedure for gastrointestinal stromal tumor (GIST) recommended by the Taiwan Surgical Society of Gastroenterology.

The TSSG recommends that patients with GIST should be managed by an MDT with expertise in sarcoma, and the recommended treatment flow is shown in Figure 2. Importantly, mutation analysis should be considered in selected patients with primary disease to confirm the diagnosis of KIT-positive GISTs with atypical morphology or clinical features, or of KIT-negative GISTs, and to identify patients at higher risk of recurrence if considering postoperative imatinib therapy after resection of the primary tumor. For the treatment of GIST, surgery remains the mainstay therapy for resectable tumors. Imatinib treatment can substantially prolong survival of patients with unresectable or metastatic GIST, and is associated with mostly mild and manageable adverse effects. Thus, imatinib should be considered as first-line treatment in metastatic GISTs. Adjuvant and neoadjuvant imatinib treatment may also be considered for patients with GIST.

Several clinical practice guidelines for GIST are now available, based on country-specific clinical practice, including those by the NCCN, ESMO, Korean GIST Study Group [58], and Japan Society of Clinical Oncology^59^. The guidelines presented here represent the updated recommendations of the TSSG for Taiwanese patients. Prepared through a series of meetings involving multidisciplinary experts across Taiwan, the recommendations have taken into account recent evidence in the diagnosis and surgical and medical treatment for GIST, and are tailored to clinical practice in Taiwan. The guidelines are intended to provide guidance for physicians in decision-making and providing optimal care and treatment for patients with GIST patients in Taiwan.

## Abbreviations

ACOSOG: American College of Surgeons Oncology Group; AFIP: Armed Forces Institute of Pathology; CT: Computed tomography; DFS: Disease-free survival; DOG-1: Discovered on GIST-1; EORTC: European Organisation for Research and Treatment of Cancer; ESMO: European Society of Medical Oncology; FDA: Food and Drugs Administration; FDG: Fluorodeoxyglucose; GIST: Gastrointestinal stromal tumors; MRI: Magnetic resonance imaging; NCCN: National Comprehensive Cancer Network; NED: no evidence of disease; NIH: National Institute of Health; OS: Overall survival; PDGFRA: Platelet-derived growth factor receptor-α; PET: Positron emission tomography; PFS: Progression-free survival; PS: Performance status; RECIST: Response Evaluation Criteria in Solid Tumors; RFS: Recurrence-free survival; RTOG: Radiation Therapy Oncology Group.

## Competing interests

The authors declare that they have no competing interests.

## Authors’ contributions

C-NY participated in the design and coordination of the study and helped to draft the manuscript; T-LH, C-SH, P-HL, C-WW, KC-G, Y-YJ and M-FC reviewed the manuscript and provided revisions. All authors have read and approved the final manuscript.
